# Aberrant Migratory Behavior of Immune Cells in Recurrent Autoimmune Uveitis in Horses

**DOI:** 10.3389/fcell.2020.00101

**Published:** 2020-03-10

**Authors:** Carmen Wiedemann, Barbara Amann, Roxane L. Degroote, Tanja Witte, Cornelia A. Deeg

**Affiliations:** ^1^Chair of Physiology, Department of Veterinary Sciences, LMU Munich, Munich, Germany; ^2^Faculty of Veterinary Medicine, Equine Hospital, LMU Munich, Munich, Germany

**Keywords:** migration, equine peripheral blood-derived lymphocytes, F-actin, septin 7, equine recurrent uveitis (ERU), autoimmune uveitis

## Abstract

The participating signals and structures that enable primary immune cells migrating within dense tissues are not completely revealed until now. Especially in autoimmune diseases, mostly unknown mechanisms facilitate autoreactive immune cells to migrate to endogenous tissues, infiltrating and harming organ-specific structures. In order to gain deeper insights into the migratory behavior of primary autoreactive immune cells, we examined peripheral blood-derived lymphocytes (PBLs) of horses with equine recurrent uveitis (ERU), a spontaneous animal model for autoimmune uveitis in humans. In this study, we used a three-dimensional collagen I hydrogel matrix and monitored live-cell migration of primary lymphocytes as a reaction to different chemoattractants such as fetal calf serum (FCS), cytokines interleukin-4 (IL-4), and interferon-γ (IFN-γ), and a specific uveitis autoantigen, cellular retinaldehyde binding protein (CRALBP). Through these experiments, we uncovered distinct differences between PBLs from ERU cases and PBLs from healthy animals, with significantly higher cell motility, cell speed, and straightness during migration of PBLs from ERU horses. Furthermore, we emphasized the significance of expression levels and cellular localization of septin 7, a membrane-interacting protein with decreased abundance in PBLs of autoimmune cases. To underline the importance of septin 7 expression changes and the possible contribution to migratory behavior in autoreactive immune cells, we used forchlorfenuron (FCF) as a reversible inhibitor of septin structures. FCF-treated cells showed more directed migration through dense tissue and revealed aberrant septin 7 and F-actin structures along with different protein distribution and translocalization of the latter, uncovered by immunochemistry. Hence, we propose that septin 7 and interacting molecules play a pivotal role in the organization and regulation of cell shaping and migration. With our findings, we contribute to gaining deeper insights into the migratory behavior and septin 7-dependent cytoskeletal reorganization of immune cells in organ-specific autoimmune diseases.

## Introduction

The research on mechanisms and motives of cells to migrate to inflammation sites through tissues and endothelial barriers is of major interest for immunologists. Therefore, a better understanding of respective signals that drive the invasion of endogenous tissue during autoimmune reactions and inflammation inside a host's body may allow early and more targeted intervention in these undesired reactions. Besides highly agile innate immune cells such as neutrophils, which act as the first line of defense at the site of inflammation (Goldberg et al., 2016), T lymphocytes are also able to achieve high-speed movements (Miller et al., 2002; Katakai and Kinashi, 2016). The exact mechanisms that enable T cells to reach high cell speed and directed migration even within dense tissue environments, however, are still elusive. The unique ameboid migration of leukocytes through dense tissue, pursuing the path of least resistance, was described to be facilitated by nuclear positioning and the ability to adapt to the physical constraints of their surrounding environment (Renkawitz et al., 2019). Also, the correlation between the cell's actin-cytoskeleton and interacting proteins plays a pivotal role during cell migration (Tooley et al., 2009; Dupré et al., 2015).

Equine recurrent uveitis (ERU) is a sight-threatening disease which affects horses worldwide (Gerding and Gilger, 2016) with a prevalence varying from 10 to 25% (Paschalis-Trela et al., 2017; Sandmeyer et al., 2017). ERU is considered an organ-specific autoimmune disease, characterized by remitting painful attacks of intraocular inflammation (Schauer et al., 2018). These inflammatory attacks are mainly driven by autoreactive CD4^+^ T cells that are somehow triggered to migrate through the blood–retinal barrier (BRB) into the inner eye, destroying intraocular structures such as the retina (Degroote et al., 2017). Increasing in severity with every attack, the relapsing inflammation eventually leads to blindness (Gerding and Gilger, 2016). An important aspect of this autoimmune disease is that autoreactive leukocytes leave the inflamed eye after first invasion, however, they reenter the eye at a later time point, causing multiple and remitting episodes of inflammation (Deeg et al., 2006a). It is of great interest to gain new knowledge on mechanisms that lead to the breakdown of immune homeostasis of the eye, as it is an immunoprivileged organ (Horai et al., 2013). In addition, ERU represents the only spontaneous animal model for human autoimmune uveitis (HAU) (Deeg et al., 2002b). Through investigations of the migratory behavior of PBLs of healthy and ERU cases, we strived to contribute to a better understanding of aberrant migration not only in this spontaneously occurring disease in horses but also, through its translational value, in uveitis of man.

Although kinetic processes and molecular mechanisms regulating T cell migration are not completely understood to date, the involved downstream mechanisms of activated T cells lead to structural reshaping of the cell, enabling their migration within the surrounding environment (Friedl and Bröcker, 2000; te Boekhorst et al., 2016; Hervas-Raluy et al., 2019). According to this, the impact of the cell's nucleus on migratory behavior during cell migration has gained importance (Hervas-Raluy et al., 2019; Renkawitz et al., 2019). We investigated possible mechanisms that control migration features of equine lymphocytes by analyzing cell movement and their ability to migrate within dense tissue through *in vitro* assays and further compared structural components interacting with the cell's cytoskeleton. Our previous findings of reduced septin 7 expression in PBLs of ERU horses (Degroote et al., 2014) prompted us to investigate septin 7 functions in equine lymphocytes and its possible contribution in the pathogenesis of ERU. Septin 7 is one of 13 members of the septin family, guanosine 5′-triphosphate (GTP)-binding and membrane-interacting proteins, being involved in various cellular processes, including cytokinesis, cytoskeleton organization, migration, and membrane dynamics (Neubauer and Zieger, 2017; Beber et al., 2019). Through interacting with actin, microtubules, and intermediate filaments, septins are described to be the fourth component of the cytoskeleton (Mostowy and Cossart, 2012). Studies on septin 7-depleted T cells showed that these cells are able to migrate through narrow pores, showing less rigidity as well as aberrant cell morphology (Tooley et al., 2009). One of the key events in the pathogenesis of ERU is the infiltration of autoreactive cells into the eye by crossing the BRB prior to an uveitic attack (Deeg et al., 2002a). Therefore, septin 7 might play an important role in this issue. We hypothesized that changes in septin 7 expression and its distribution and interaction with actin structures might contribute to an altered migratory behavior of PBLs of ERU cases.

## Materials and Methods

### Isolation of Primary Peripheral Blood-Derived Lymphocytes

For all experiments within this study, we sampled a total of 14 different controls and 17 different ERU cases. Samples from some of the animals were used in multiple assays. In detail, PBLs of 10 controls and 11 ERU cases were used for live cell experiments with fetal calf serum (FCS) as a chemoattractant. PBLs of four controls and four ERU cases were examined in live-cell experiments using interleukin-4 (IL-4), interferon-γ (IFN-γ), and cellular retinaldehyde binding protein (CRALBP) as source of chemoattractant. PBLs from three healthy controls were treated with forchlorfenuron (FCF) and used for migration assays on glass slides. PBLs of eight controls and eight ERU cases, which have also been used in live-cell experiments, were used for quantification of septin 7 expression by Western blot. In addition to biological replicates, at least three technical replicates were used per animal within different experiments. At the time of blood withdrawal, ERU cases were all in a quiescent stage. ERU was diagnosed by experienced clinicians from the Equine Hospital of LMU Munich and was based on typical clinical signs of uveitis along with a documented history of multiple episodes of inflammation of the affected eye (Brandes et al., 2007). Only eye-healthy horses without any signs of disease, which were regularly monitored by veterinarians, served as controls. Equine venous blood samples were taken from the *vena jugularis*. The collection of blood samples was permitted by the ethical committee of the local authority (Regierung von Oberbayern; permit number: ROB-55.2Vet-2532.Vet_03-17-88). Blood was collected in tubes with heparin sodium (50 IU/ml blood; Ratiopharm, Ulm, Germany). After sedimentation of the blood component, the lymphocyte-rich plasma was isolated by density gradient centrifugation [room temperature (RT), 350 × g, 25 min, brake off] using Pancoll separation solution (PanBiotech, Aidenbach, Germany). Lymphocytes were extracted from the intermediate phase, washed three times in phosphate buffered saline (PBS), and kept in cell culture medium at 1 × 10^6^ cells/ml, without supplementing any serum, overnight.

### *In vitro* Migration Assays

Live-cell chemotaxis assays within a three-dimensional (3D) collagen matrix were performed using μ-slides chemotaxis 3D (ibidi, Gräfelfing, Germany), with its compatible heating and incubation system (ibidi Stage Top Incubation Systems, Multiwell-Plates, K-Frame). These μ-slides are well-established and calibrated by the manufacturer and provide a quick and time-stable gradient to investigate chemotaxis and migratory behavior of adherent or suspension cells, within 2D or 3D surroundings (Zengel et al., 2011; Biswenger et al., 2018). The μ-slide is built of a 70 × 1,000 μm-wide channel, which is connected to two chemoattractant-filled chambers, to attain a stable gradient throughout the channel. A microscope with automated time-lapse features was used for observation of live-cell migration assays (Leica Dmi8, Leica Microsystems, Wetzlar, Germany). Prior to chemotaxis live-cell experiments, the required materials and cells were stored for at least 8 h within an environment that featured 37°C and 5% CO_2_ to ensure adequate gas equilibration and to facilitate quality and success of live-cell experiments using μ-slides. To create a 3D matrix within the channels of the μ-slides, the final collagen concentration (1.5 mg/ml) and cell concentration (1.8 × 10^8^ cells/ml) within the final volume of 150 μl collagen solution were calculated. According to the manufacturer's instructions, all ingredients were stored on ice and mixed as listed in the application note [ibidi, AN26 (https://ibidi.com/img/cms/support/AN/AN26_CollagenI_protocols.pdf)]. In detail, 10 μl of 10× Dulbecco's modified Eagle's medium (DMEM) low glucose (10× DMEM-low glucose; Sigma-Aldrich/Merck, Darmstadt, Germany), 2.5 μl 1 M NaOH (Applichem, Darmstadt, Germany), 40.5 μl distilled water, 2 μl NaHCO_3_ 7.5% (Sigma-Aldrich/Merck), and 25 μl DMEM (PanBiotech) were prepared within a sterile tube. Lastly, 45 μl of 5 mg/ml collagen rat tail type I (ibidi) and 25 μl containing 4.5 × 10^5^ PBLs were added to the tube and mixed thoroughly. For polymerization of the collagen-cell solution, μ-slides were placed in a cell culture incubator (37°C, 5% CO_2_) for 30 min. Throughout all experiments, no supplemental serum was added to the collagen gel, independently of the source of chemoattractant. Afterward, both reservoirs of the μ-slides were filled with medium on one side and medium plus chemoattractant on the other side, respectively. Time-lapse imaging of immune cell migration within μ-slides was performed for 3 h, taking one image every 60 s of each observation channel containing cells, with a Leica Dmi8 microscope. Within these assays, FCS (Biochrom, Berlin, Germany) (20% FCS solution), equine IL-4 (10 ng/ml), equine IFN-γ (10 ng/ml) (both Biomol, Hamburg, Germany), and CRALBP [0.1 mg/ml, as an organ-specific autoantigen, self-made (Deeg et al., 2006b)] were used as chemoattractants. The chemoattractants were diluted with DMEM, and 30 μl of the respective solutions were inserted in the bottom reservoir of the μ-slides. Positive cell responses and effects to respective attractants led to a shift of the cell population downward, toward the source.

### Alteration of Cellular Septin 7 Scaffolding Through Forchlorfenuron

One hour prior to carrying out live-cell experiments or examining protein expressions through immune cytology after migration on glass slides, cells were incubated with the synthetic plant cytokinin N-(2chloro-4-pyridyl)-N9-phenylurea, known as FCF (Sigma-Aldrich/Merck). Due to the fact that FCF impairs septin structures by dampening septin filaments, leading to elongated, and more densely packed structures (Hu et al., 2008), we aimed to investigate the effects of this cytokinin on migratory behavior of equine PBLs. Within a sterile tube, 50 μl cell suspension containing 9 × 10^5^ PBLs of healthy controls were incubated for 1 h with 50 μM FCF, diluted in DMEM, and experiments proceeded as described.

### Image Analysis

Image stacks of time-lapse recordings of all channels and slides were exported as image data (.tif). We used the open-source ImageJ software (https://imagej.net/), with the manual tracking plugin for ImageJ (https://imagej.net/Manual_Tracking), to track immune cells within the 3D collagen matrix inside the channels of ibidi μ-slides. For objective analysis and reliable results of migration assays, all cells were randomly chosen in the observation channel and not preselected for any of the parameters. After tracking of at least 20–30 single cells per channel, direction, distance, and velocity of the cells were analyzed using the Chemotaxis and Migration Tool Version 1.01 (https://ibidi.com/chemotaxis-analysis/171-chemotaxis-and-migration-tool.html).

### Immunocytology of Blood-Derived Lymphocytes Migrating on Glass Slides

For these experiments, we used untreated and FCF-treated equine PBLs (1.8 × 10^7^ cells/ml), diluted in DMEM. A volume of 25 μl of cell-dense suspension was applied onto the middle of glass slides, nearby the source of chemoattractant (FCS, neat). The glass slides were located on a compatible rack within the incubation and heating chamber of the ibidi system (37°C, 5% CO_2_). After cells moved for 10 min, glass slides were immediately fixed with ice-cold aceton for 10 min. Afterward, slides were rehydrated in suitable buffer, Tris buffered saline with Tween 20 (TBS-T), for 15 min and blocked with 1% bovine serum albumin (BSA) (Applichem) for another 45 min. Cells were stained overnight with antibodies, namely, anti-equine septin 7 (rat monoclonal IgG2c, fluorescein isothiocyanate (FITC) conjugated, 1:1,000, self-made) and anti-phalloidin for staining F-actin filaments in the cytoskeleton [tetramethyl rhodamine isothiocyanate (TRITC) conjugated, Sigma-Aldrich/Merck; 1:100]. Cell nuclei were counterstained with 4′,6-diamidino-2-phenylindole (DAPI) (Invitrogen; 1:100). Slides were washed three times within TBS-T for 10 min and dried after applying cover glasses. Pictures of respective glass slides were taken with a Leica Dmi8 microscope. High-resolution images were performed with the same microscope, using a 100× - magnification objective. Differences of fluorescent signals were examined and analyzed with LAS-X software from Leica (https://www.leica-microsystems.com/products/microscope-software/).

### Quantification of Septin 7 Expression by Western Blot

PBLs of healthy horses and ERU cases were lysed in lysis buffer [9 M urea, 2 M thiourea, 65 mM dithioerythritol, 4% 3-((3-cholamidopropyl) dimethylammonio)-1-propanesulfonate (CHAPS)]. Subsequently, proteins were separated by sodium dodecyl sulfate (SDS)-polyacrylamide gel electrophoresis (PAGE) on 8% gels (7 μg protein/slot) and blotted semidry onto 8.5 × 6 cm polyvinylidene fluoride (PVDF) membranes (Carl Roth, Karlsruhe, Germany). Unspecific binding was blocked with 4% bovine serum albumin for 1 h at room temperature. Blots were incubated with horseradish peroxidase (HRP)-coupled anti-equine septin 7 [rat monoclonal IgG2c, 1:10,000, self-made (Schauer et al., 2018)] at 4°C overnight. After six washing steps, signals were detected by enhanced chemiluminescence using Amersham Imager600 (GE Healthcare, Freiburg) for developing respective membranes. To control equal loading of respective protein samples, the antibody mouse anti-beta actin (Merck, Darmstadt, Germany; 1:500,000) was incubated overnight at 4°C. Blots were washed three times with PBS with Tween 20 (PBS-T) between antibody incubation steps. HRP-coupled anti-mouse IgG antibody (Merck, Darmstadt, Germany; 1:5,000) was incubated for 1 h at room temperature. After six washing steps, signals were detected by enhanced chemiluminescence as previously described. Quantification of septin 7 signals was achieved by using open-source ImageJ software (https://imagej.net/). All septin 7 signals were normalized to respective beta actin signals. Septin 7 signals from ERU samples were compared to healthy controls with statistical analysis using Student's *t*-test, and intensity differences were considered significant at *p* < 0.05.

### Statistical Analysis

The Kolmogorow–Smirnov (KS) test was used for determination of Gaussian distribution. If KS test was significant (*p* < 0.05; no normal distribution), Mann–Whitney test was used for statistical analysis; if KS test was not significant (*p* > 0.05; normal distribution), statistics were performed using Student's *t*-test. In both tests, statistical probabilities were considered significant at *p* < 0.05. Significances are indicated by asterisks with ^*^p < 0.05, ^**^p < 0.01, and ^***^p < 0.001.

## Results

### Equine Recurrent Uveitis Lymphocytes Migrated More Directed and Faster Toward Fetal Calf Serum

**Figure 1:**
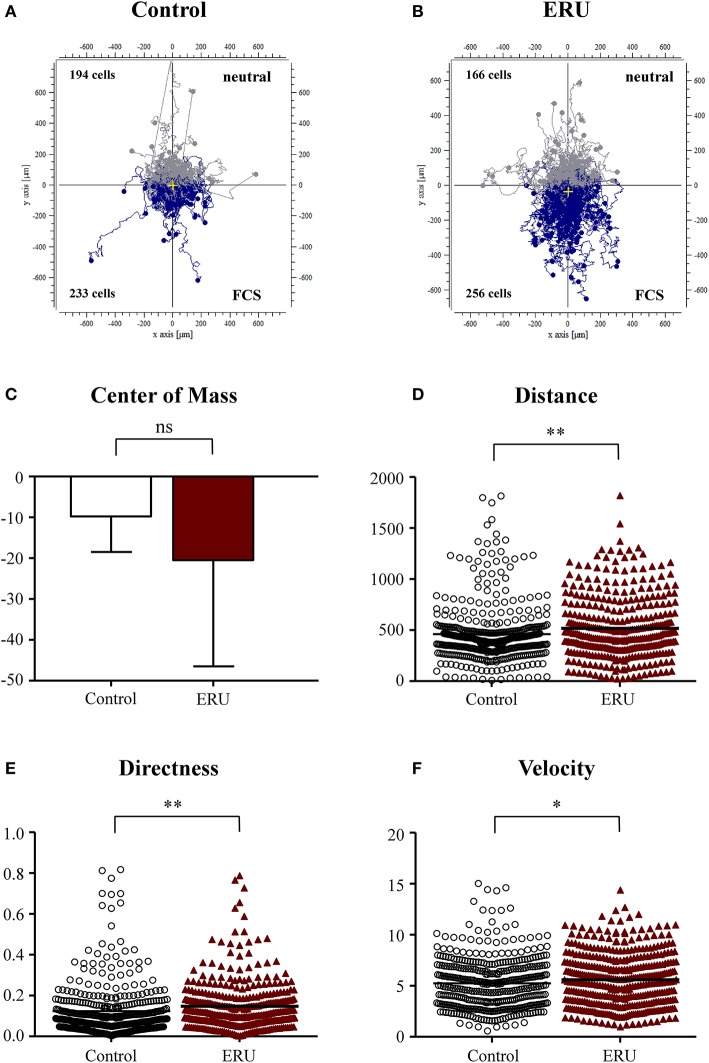
**(A,B)** showing cell trajectory plots of μ-slide chemotaxis live-cell experiments using fetal calf serum (FCS) as the source of chemoattractant with tracked peripheral blood-derived lymphocytes (PBLs) of 10 controls and 11 equine recurrent uveitis (ERU) cases. Starting points of each cell were placed in the center of the plots. Cells moving toward FCS were marked in blue; cells that were not attracted by the source were marked in gray. Cell numbers inside the plots represent tracked cells of either PBLs from controls or ERU cases and show amount of cells that migrated up (away from source) or down (toward the source). No differences were analyzed by comparison of values of the center of mass **(C)**. Migration parameters of cells from ERU cases revealed increased traveled distance **(D)** (**p < 0.01), directness **(E)** (**p < 0.01), and velocity **(F)** (*p < 0.05). Dots or triangles within graphs D to F represent individual cells. Horizontal lines correspond to mean values.

Within the live-cell experiments in the 3D system, we were able to observe and analyze distinct cell migration throughout all conducted assays. First, we used FCS as an attractant to test migration abilities of primary equine immune cells of healthy and ERU cases. We verified and compared the migration parameters displacement of the center of mass, distance (μm), directness, and velocity (μm/min). In total, 427 cells from healthy controls and 422 cells from ERU cases have been tracked throughout live-cell experiments using FCS as chemoattractant. Cell trajectories revealed that a higher amount of ERU immune cells, 256 cells, compared to 233 cells of controls, moved toward FCS (Figures 1A,B). The displacement of the center of mass of ERU cells, which represents the whole migrated cell population, shifted closer to the site of attractant than the controls (Figure 1C). Further, immune cells of ERU cases showed significantly longer migration distances throughout the experiments. Cells of ERU cases migrated 507 μm on average, cells from controls 460 μm (^**^p < 0.01; Figure 1D). Besides this, cells of ERU cases moved more directed during the time period of live-cell experiments (^**^p < 0.01; Figure 1E), which represents the straightness of the cell's path from their starting to their endpoint. Additionally, immune cells derived from ERU cases migrated significantly faster through the collagen-dense matrix toward the attractant site, moving 5.6 μm/min on average while cells of controls moved 5.2 μm/min (^*^p < 0.05; Figure 1F).

### Chemotactic Effect Does Not Occur With Interleukin-4 as Source of Attractant

**Figure 2:**
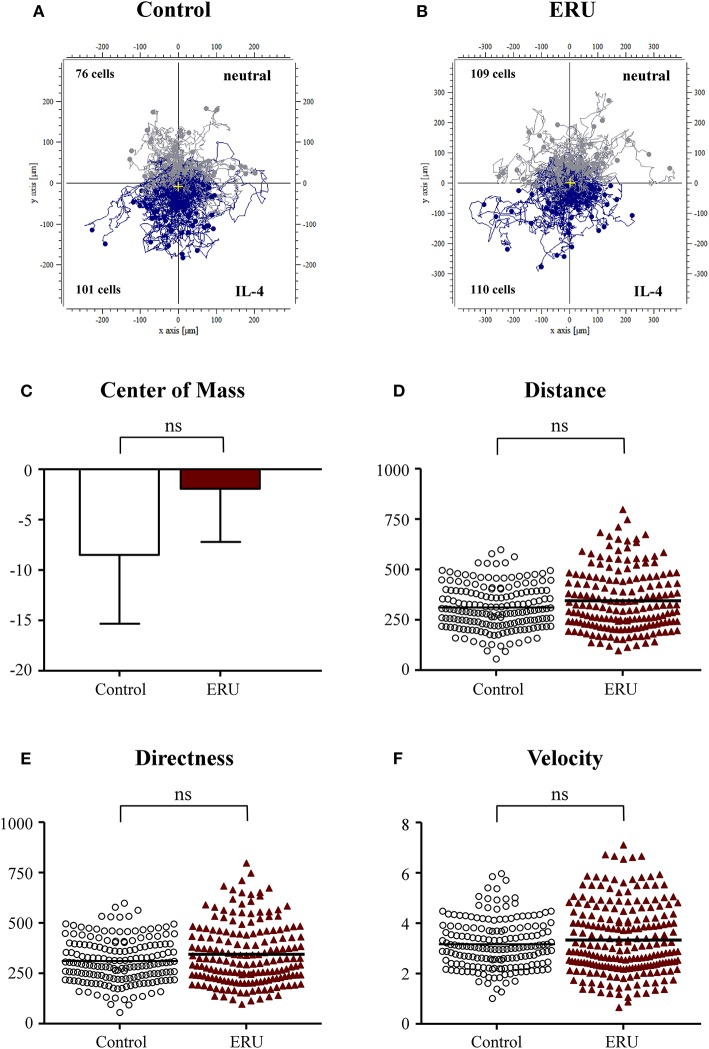
Cell trajectory plots of μ-slide chemotaxis live-cell experiments of peripheral blood-derived lymphocytes (PBLs) from four healthy horses **(A)** and PBLs from four equine recurrent uveitis (ERU) cases **(B)** using interleukin-4 (IL-4) as the source of chemoattractant. Starting points of each cell were placed in the center of the plots. Tracks illustrate cells moving toward IL-4 (blue) or away from the source of chemoattractant (gray). Cell numbers inside the plots show the amount of cells that moved up (away from source) or down (toward the source of chemoattractant). No significant differences in migration parameters displacement of the center of mass **(C)**, distance **(D)**, directness **(E)**, and velocity **(F)** were analyzed between PBLs from controls and ERU cases (not significant, *p* > 0.05). Dots or triangles within graphs **(D–F)** represent individual cells. Horizontal lines correspond to mean values.

Besides using FCS as the source of attractant, we investigated differences in migration parameters using signature cytokines IL-4 and IFN-γ. There were no significant differences in migration parameters of the 177 tracked immune cells from healthy cases and the 219 tracked cells from ERU cases (Figures 2A–F). Cell trajectory plots showed a similar appearance, as nearly the same amount of healthy and ERU cells moved toward or away from the source of chemoattractant (Figures 2A,B). No significant differences regarding migration distance, displacement of the center of mass, directness, or velocity were detected (not significant, *p* > 0.05; Figures 2A–D).

### Interferon-γ Induces Higher Cell Velocity and Migration Distance of Equine Recurrent Uveitis Immune Cells

**Figure 3:**
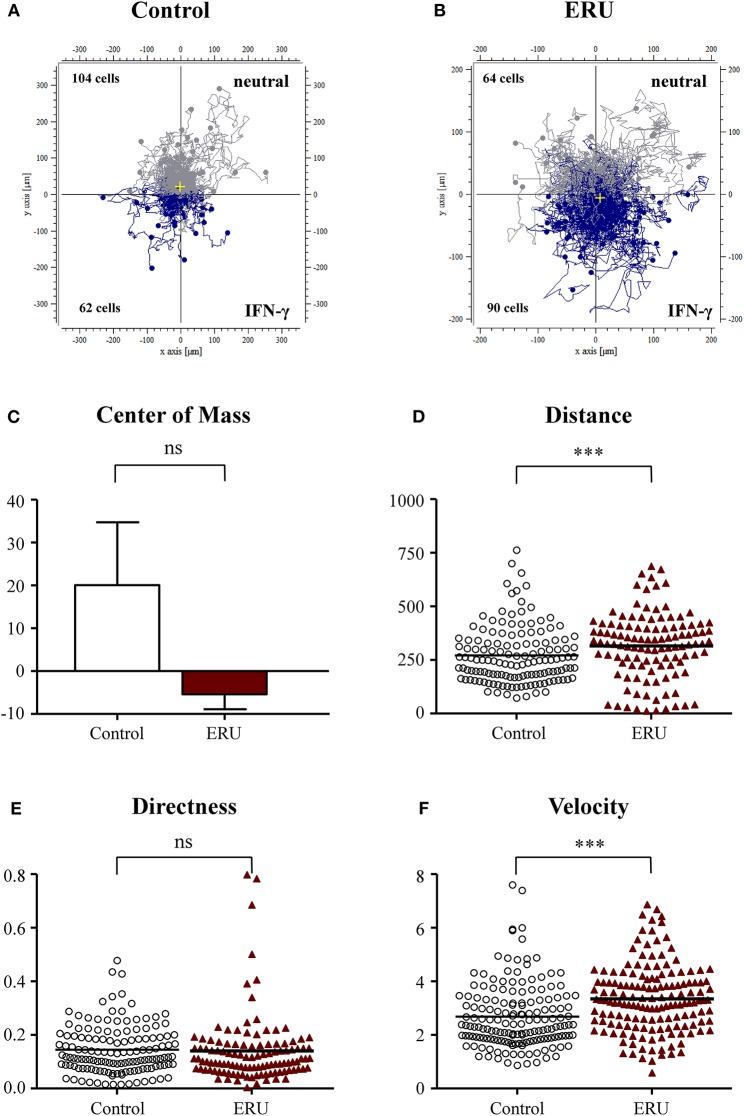
Cell trajectory plots of lymphocytes from four healthy **(A)** and four equine recurrent uveitis (ERU) cases **(B)** with interferon-γ (IFN-γ) as the source of chemoattractant. Starting points of each cell were placed in the center of the plots. Blue tracks illustrate cells moving toward IFN-γ; gray tracks show cells not attracted by the source. The displacement of the center of mass **(C)** showed that more than 50% of control peripheral blood-derived lymphocytes (PBLs) moved in the opposite direction of IFN-γ. Analysis of migration parameter values of PBLs from controls and ERU cases revealed significantly greater traveled distances **(D)** (***p < 0.001), no differences in directness **(E)**, but significant faster migration **(F)** (***p < 0.001) of cells from ERU cases. Dots or triangles within graphs **(D–F)** represent individual cells. Horizontal lines correspond to mean values.

The chemokine IFN-γ plays a pivotal role in the emergence of ERU, an autoimmune disease with Th1-immune response (Gilger and Deeg, 2011). Since ERU is a Th1-dependent disease, we next tested the impact of this signature cytokine on migratory behavior of lymphocytes of controls and ERU cases. Cells of healthy controls did not respond to the application of IFN-γ since two thirds of migrated healthy immune cells (104 out of 166 tracked cells) were moving away from the site of attractant (Figure 3A). This result could also be clearly seen by the center of mass analysis (Figure 3C). PBLs of ERU horses, on the other hand, showed higher response and stronger movement toward the reservoir filled with IFN-γ, as almost two thirds of all tracked cells (90 out of 154 tracked cells) moved downstream to the site of attractant (Figures 3B,C). Furthermore, ERU immune cells migrated significantly longer distances within the 3D matrix, in detail migrating 305 μm, compared to controls with 255 μm on average (^***^p < 0.001; Figure 3D). However, there were no significant differences in the directness of migrating cells (Figure 3E). PBLs from ERU cases showed a highly significant distinction in terms of cell speed through dense collagen tissue, as ERU cells moved with an average of 3.3 μm/min, whereas control cells migrated with 2.6 μm/min on average (^***^p < 0.001; Figure 3F).

### Equine Recurrent Uveitis Immune Cells Show a Significantly Higher Response and Migratory Behavior to Autoantigen Cellular Retinaldehyde Binding Protein

**Figure 4:**
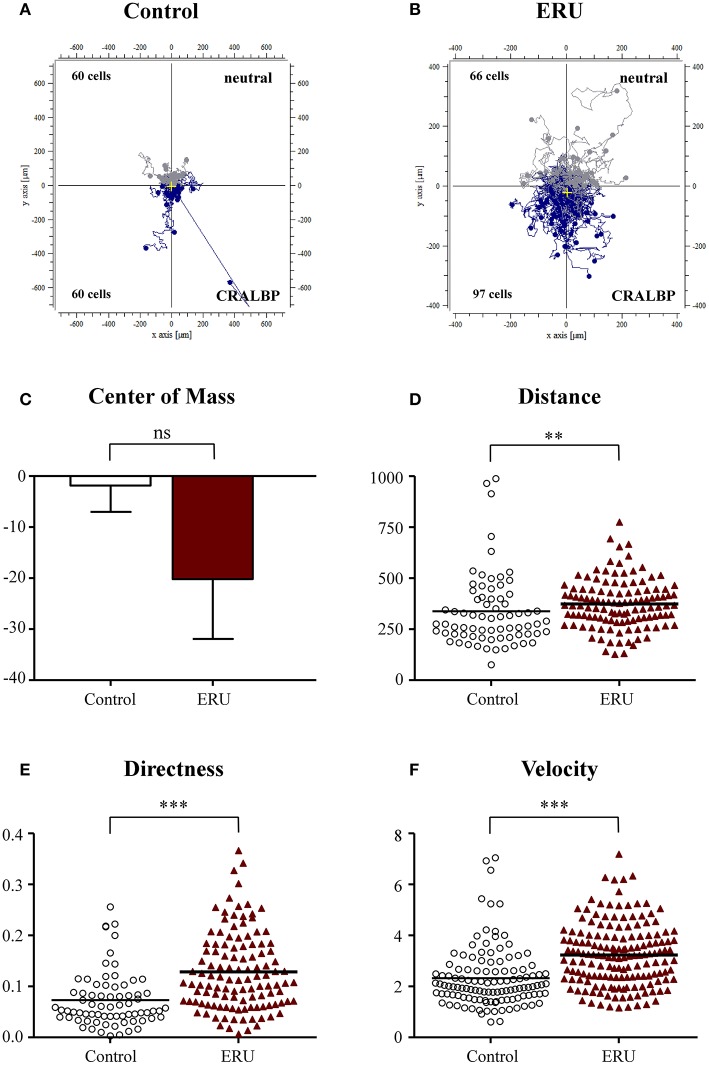
Cell trajectory plots of peripheral blood-derived lymphocytes (PBLs) from healthy **(A)** (*n* = 4) and equine recurrent uveitis (ERU) cases **(B)** (*n* = 4) using retinal autoantigen cellular retinaldehyde binding protein (CRALBP) as attractant. Starting points of each cell were placed in the center of the plots. Blue tracks illustrate cells moving toward, gray tracks show cells moving away from source. Cell numbers inside the plots represent tracked cells of controls or ERU cases that migrated up (away from source) or down (toward source). Comparison of migration parameters from ERU cases and controls uncovered no differences between values of the center of mass **(C)**, great differences in traveled distances **(D)** (**p < 0.01), as well as significantly more directed **(E)** and faster migration **(F)** (***p < 0.001) of cells from ERU cases. Dots or triangles within graphs **(D–F)** represent individual cells. Horizontal lines correspond to mean values.

We next applied CRALBP in our live-cell experiments and used this autoantigen as an autoimmune-specific target for immune cells of ERU horses. CRALBP is an autoantigen which was proven to be relevant in the pathogenesis of ERU and which also plays a role in autoimmune uveitis in man (Deeg et al., 2006b, 2007). PBLs of healthy horses did not show high responsiveness toward the site of this autoantigenic attractant. Namely, 60 out of 120 tracked cells moved toward the reservoir filled with CRALBP, while the other half moved into the opposite direction (Figure 4A). In contrast, two thirds of 163 tracked ERU cells, 97 in detail, moved significantly closer toward the reservoir containing CRALBP (Figures 4B,C). ERU cells also migrated longer distances within the collagen matrix, moving 343 μm, compared to an averaged distance of 287 μm of the controls (^**^p < 0.01; Figure 4D). Additionally, they showed clearly directed migration (^***^p < 0.001; Figure 4E) in comparison to the cells of healthy controls. Further, control cells moved significantly slower (with 2.3 μm/min) than immune cells of ERU cases with an average of 3.3 μm/min (^***^p < 0.001; Figure 4F). These results represent a highly significant and specific responsiveness of PBL of ERU cases toward this autoantigen.

### Impairment of Septin 7 Scaffolding Structures Through Forchlorfenuron Alters Migration of Lymphocytes

**Figure 5:**
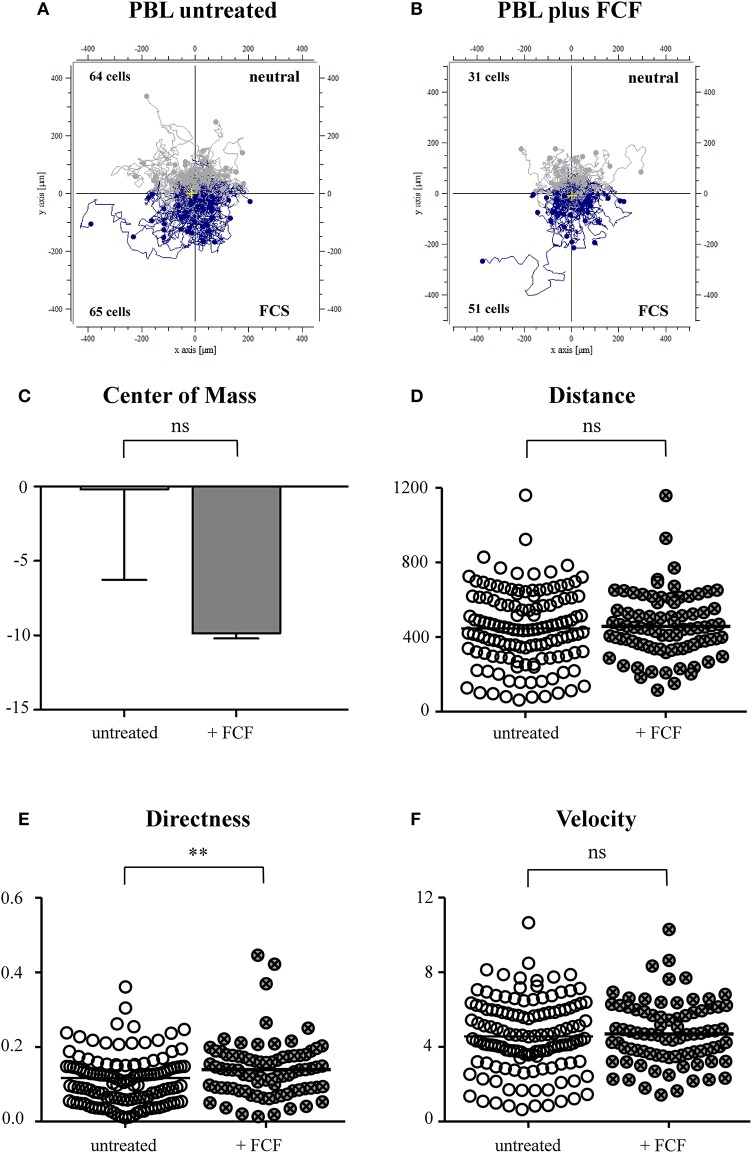
Cell trajectory plots of untreated **(A)** and forchlorfenuron (FCF)-treated **(B)** peripheral blood-derived lymphocytes (PBLs) of healthy horses. Starting points of each cell were placed in the center of the plots. Blue tracks illustrate cells moving toward the site of attractant (FCS, fetal calf serum); gray tracks show cells moving away from source. The numbers inside the plots represent tracked cells of either PBLs of untreated or FCF-treated controls and show numbers of cells moving up (away from source) or down (toward source of chemoattractant). Analysis of migration parameters revealed that there were no significant differences between values of untreated and FCF-treated cells in the center of mass **(C)** and the distance **(D)**. FCF-treated cells showed a significantly more directed movement **(E)** (**p < 0.01); however, there were no differences between velocity parameters **(F)**. Dots within graphs **(D–F)** represent individual cells. Horizontal lines correspond to mean values.

Based on our results from the live-cell experiments, we were interested in mechanisms and proteins enabling immune cells of ERU horses to migrate faster and more directed through dense collagen tissue than healthy PBLs. The interdependence of septin structures with the actin cytoskeleton plays a major role when contemplating migration and motility of lymphocytes (Schmidt and Nichols, 2004; Lam and Calvo, 2019). As PBLs of ERU cases showed less septin 7 expression (Degroote et al., 2014), we supposed that impairment of septin 7 structures of healthy PBLs might result in similar migratory behavior as ERU PBLs. To show possible impacts of migratory behavior and parameters due to impairment of septin 7 and interacting structures, we set up an experiment including FCF-treated cells and a control group with untreated PBL from the same individual. By comparing migration trajectories of untreated to FCF-treated PBLs, only slight differences were obtained (Figures 5A,B). In relation to the migrated cell number, a higher amount of FCF-treated PBLs moved toward the source of FCS (Figure 5B). The displacement of the center of mass of FCF-treated PBLs shifted more to the attraction site, however not statistically significant (*p* > 0.05; Figure 5C). There was also no significant distinction between untreated and FCF-treated PBLs concerning the migrated distance (Figure 5D). However, FCF-treated cells showed significantly more directed movement through dense collagen tissue toward the attractant site (^**^p < 0.01; Figure 5E). FCF treatment did not affect cell velocity of respective cells (not significant, *p* > 0.05; Figure 5F).

### Migratory Behavior Associated With Changed, Intracellular Distribution of F-Actin and Septin 7 in Equine Peripheral Blood-Derived Lymphocytes

**Figure 6:**
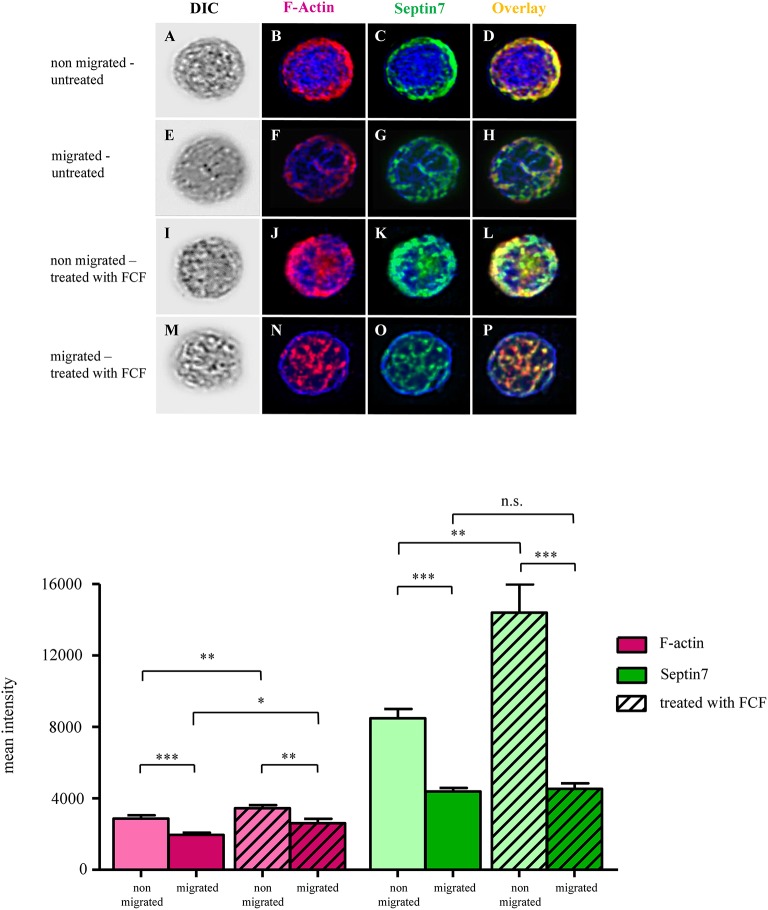
Representative images of untreated and forchlorfenuron (FCF)-treated equine lymphocytes of a healthy control. First column of each row shows differential interference contrast (DIC) images of lymphocytes **(A,E,I,M)**. F-actin is shown in magenta (**B, F, J, N**: single channel; **D,H,L,P**: overlay), septin 7 expression is displayed in green (**C,G,K,O**: single channel; **D,H,L,P**: overlay). Cell nuclei were stained with 4′,6-diamidino-2-phenylindole (DAPI; blue). Untreated, non-migrated lymphocytes had densely packed F-actin and septin 7 structures along the cell's plasma membrane **(B–D)**. F-actin structures of untreated, migrated cells **(F)** were less intense and more delicate. Septin 7 filaments were less dense and expressed at the plasma membrane and perinuclear regions **(G)**. F-actin and septin 7 structures of non-migrated, FCF-treated cells were densely packed, mainly distributed along the cell's plasma membrane **(J–L)**. F-actin and septin 7 structures of migrated, FCF-treated lymphocytes were distributed more evenly throughout the cell, and more expressed in perinuclear regions, with minor association to the plasma membrane **(N–P)**. Mean intensities of F-actin were significantly reduced in untreated, migrated cells (***p < 0.001; first two magenta bar graphs), and F-actin signal was significantly reduced in FCF-treated cells that were migrated (**p < 0.01; magenta bar graphs with diagonal lines). Mean intensities of F-actin were significantly higher in migrated FCF-treated cells, compared to untreated cells that migrated (*p < 0.05; right magenta bars with and without diagonal lines). Septin 7 was significantly reduced in control peripheral blood-derived lymphocytes (PBLs) that migrated in contrast to cells that did not migrate (***p < 0.001; first green bar graphs). Septin 7 signal was also significantly reduced in FCF-treated and migrated cells compared to respective non-migrated cells (***p < 0.001; outer green bar graphs with diagonal lines).

Intracellular localization and composition of respective proteins can also have great impact on cell behavior regarding adaption of cell shape and movement (Dunn et al., 2011). Through their scaffolding role inside cells, septins function as membrane-associated and signaling proteins (McQuilken et al., 2017; Lam and Calvo, 2019). Therefore, we conducted an analysis of protein distributions within untreated and FCF-treated PBLs, comparing non-migrated to migrated PBLs using differential interference microscopy (Figures 6A,E). Non-migrated, untreated lymphocytes showed densely packed F-actin (Figure 6B) and septin 7 (Figure 6C) structures. These structures were mainly located at the plasma membrane. Overlay of both channels demonstrates nearby localization of both structures (Figure 6D). In contrast to non-migrated cells, migrated PBLs showed widely spread and more delicate F-actin (Figure 6F) and septin 7 structures (Figure 6G). The distribution of respective proteins reached from the plasma membrane more toward the cell's nucleus (Figure 6H). In addition, we investigated FCF-treated PBLs through differential interference contrast (DIC) microscopy (Figures 6I,M). Non-migrated PBLs treated with FCF showed thicker, more densely packed structures of F-actin (Figures 6J,L) and septin 7 (Figures 6K,L). Respective structures were closely located at the plasma membrane and showed increased and condensed distribution toward the cell's nucleus (Figures 6J–L). Migrated FCF-treated PBLs represented a continuous distribution of F-actin and septin 7 structures, being more subtle (Figures 6N–P). Further, respective proteins switched from marginal localization along the plasma membrane toward nuclear regions (Figures 6N–P).

### Migrated Lymphocytes Had Reduced F-Actin and Septin 7 Mean Intensities Compared to Non-migrated Cells

Since FCF-treated lymphocytes of healthy individuals showed increased directness, we were interested in possible differences in the expression and distribution of F-actin and septin 7 of non-migrated and migrated PBLs, either untreated or treated with FCF. For each condition, at least 35 cells were analyzed for their mean intensity of F-actin and septin 7 (representative images of lymphocytes; Figures 6A–P). The quantified signals of F-actin in untreated, migrated lymphocytes was significantly reduced (^***^p < 0.001; Figure 6, magenta bars). Further, FCF-treated, migrated PBLs expressed less F-actin compared to FCF-treated, non-migrated PBLs (^**^p < 0.01; Figure 6, magenta bars with diagonal lines). The mean intensity of septin 7 expression in untreated, migrated PBLs revealed a significant two-fold lower expression of septin 7 as in non-migrated PBLs (^***^p < 0.001; Figure 6, green bar graphs). FCF-treated, migrated PBLs expressed only a third of septin 7 compared to FCF-treated, non-migrated cells (^***^p < 0.001; Figure 6, green bars with diagonal lines). Taken together, migrated PBLs showed significantly decreased mean intensities of both F-actin and septin 7 compared to non-migrated cells in untreated lymphocytes.

## Discussion

A key event in the pathogenesis of ERU is the migration of autoreactive blood-derived leukocytes over the BRB, attacking and destroying retinal tissue in the immune privileged eye (Degroote et al., 2017; Schauer et al., 2018). In this study, we aimed to increase knowledge about migratory behavior and underlying molecular mechanisms of equine lymphocytes in ERU. For this purpose, we investigated migration parameters of primary lymphocytes (Supplemental Figure 1) as well as the role of septin 7 and F-actin, which possibly participate and promote migration of respective cells.

Our initial findings on migratory behavior showed that PBLs of ERU cases migrated greater distances and moved significantly faster through dense collagen matrix compared to PBLs of healthy animals, increasing their cell speed by up to 29% (Figures 1–4). Higher cell migration speed and traveled distance in ERU seems to be independent of chemoattractant used, since this occurred in all experiment setups, except for chemoattractant IL-4, which was used as control (Figure 2). Hence, ERU cells seem to be more active in terms of cytokinesis, readily awaiting migration as cytokines are introduced. From other studies on interstitial migration of T cells in mouse models we know, that higher migration velocities are linked to the activation status of cells as well as their responsiveness to inflammatory cytokines (Katakai et al., 2013). Therefore, we hypothesized that a higher mean velocity of cells from ERU cases could indicate an increased amount of activated cells within ERU PBL population. Currently, we do not know the exact mechanisms driving PBLs from ERU cells to migrate faster and further. However, this feature of ERU PBLs supports data on T cells being activated and primed for intraocular structures in the periphery—for yet unknown reasons—and subsequent relocation from the periphery to the target organ, the immune privileged eye (Gilger and Deeg, 2011). In correlation with this, the biggest difference in velocity between healthy animals and ERU horses was detected with IFN-γ, a cytokine which shows high abundance in the vitreous of ERU horses, and CRALBP, a specific ocular autoantigen which is highly expressed in ERU rentinae (Figures 3, 4). The high responsiveness of PBLs of ERU cases to these substances points to the presence of activated, pro-inflammatory Th1 cells within the migrating cell population of ERU horses. Evidence of Th1 cells driving pathogenesis-associated mechanisms in autoimmune diseases such as ERU have previously been proven in several studies, revealing increased transcription of IFN-γ in the vitreous as well as higher levels of IFN-γ expression in PBLs from ERU horses (Gilger et al., 1999; Saldinger et al., 2019). CRALBP additionally caused a more directed migration of ERU cells (Figure 4). In our equine model, we do not know the exact cause of increased directness of PBLs from ERU cases so far. However, migration experiments with murine T cells showed that directed migration can be associated with higher velocity, which correlates with the activation status of the cells, resulting in better adaptation of cell shape within extracellular surroundings (Lammermann and Kastenmuller, 2019). We suggest that velocity and directness might both correlate with a higher amount of activated cells in PBLs of ERU cases, enabling a better migration of these cells within dense matrices through more effective adaption of cell morphology. To assess this issue in future studies, we will investigate migratory behavior of PBLs from healthy controls after targeted activation through specific T-cell stimulants.

Among whole PBL populations in ERU horses, autoreactive precursors are scarce, especially in the quiescent stage of the disease (Deeg et al., 2002b, 2004, 2006a). However, this small amount of autoaggressive T cells will suffice to trigger inflammation of the inner eye, as proven in adoptive transfer studies of experimental autoimmune uveitis (EAU) rodent models, where as little as 15 primed cells were sufficient to efficiently induce an uveitic attack (Caspi, 2006). Although the detected differences in migratory behavior in our studies may seem quite subtle, they are surprisingly clear, considering given sample features: neither were sampled horses preselected for especially high amounts of autoreactive T cells, nor were analyses performed on preselected T cell populations or isolated autoaggressive precursor cells. In addition, individual variation due to the use of biological rather than technical replicates and the naturally low amount of primed T cells in our PBL samples contribute to less apparent differences. But even in this context, our data show significant differences in traveled distance, directness, and velocity of ERU cells, even in the quiescent stage of the disease, pointing to subsisting and continuous presence of autoreactive cells between attacks. Furthermore, the detected migration differences in ERU cells and their association to a Th1 response correlate with the predominant Th1 cell phenotype involved in ERU (Gilger et al., 1999; Saldinger et al., 2019). Data from other models show similar changes in migratory parameters. In a mouse model for autoimmune diabetes, interstitial migration of T cells was investigated within pancreatic tissue (Espinosa-Carrasco et al., 2018). T cells, which migrated in islets of exocrine pancreas in control animals migrated with an average velocity of 3.2 μm/min compared to T cells within anti-CXCR3-treated mice with 4 μm/min, which was considered as highly significant (^****^p < 0.0001; Espinosa-Carrasco et al., 2018). The same applies to averaged velocities in exocrine pancreatic tissue, where T cells within control mice migrated approximately 6.5 μm/min compared to T cells within anti-CXCR3-treated mice with an averaged velocity of ~6.0 μm/min (Espinosa-Carrasco et al., 2018). Thus, migration velocities of T cells within islets or exocrine pancreatic tissue of mice that were pretreated with anti-CXCR3 antibody migrated roughly 10% faster compared to T cells in mice that were treated with isotype control antibody (Espinosa-Carrasco et al., 2018).

Although studies on migration of T cells exist, these are mostly done with pretreated cells, established cell lines, or cells from artificially induced animal models (John et al., 2009; Tooley et al., 2009; Cai et al., 2019). Comparable migration studies, using primary, non-preselected PBLs from a spontaneously occurring, organ-specific autoimmune disease, however, are scarce to date. Therefore, with our data, we provide an initial basis, which can be expanded through presorting of autoimmune precursor cells for migration assays and more in-depth analyses in order to clarify the exact meaning of the migration differences we detected in our studies.

As regulation and signaling pathways in migration of equine leukocytes are still to be uncovered, we next aimed at finding possible contributing mechanisms on protein level. Since we previously detected lower septin 7 expression in PBLs of ERU cases (Degroote et al., 2014) and we also found decreased septin 7 levels in the ERU cells used in our migration assays (Supplemental Figure 2), we were interested if this decrease correlates with changed migration behavior in these cells. Septin 7 is strongly associated to cytoskeletal structures such as actin and tubulin and might therefore play a pivotal role in cytokinesis and migration (Mostowy and Cossart, 2012). Interestingly, in murine T cells, septin 7 decrease affects plasticity and deformability, allowing migration through very narrow pores (Tooley et al., 2009). Based on these findings, we suggest that equine cells with decreased septin 7 expression might have increased capability of overcoming the BRB, which physiologically acts as a barrier to prevent immune cells from entering the eye through changes in cytoskeletal structures.

To further investigate the possible contribution of septin 7 expression levels to migration of equine T cells, we used FCF, a synthetic plant cytokinin, which reversibly alters septin assembly and dynamics, but has no effect on actin and tubulin polymerization or viability of cells (Hu et al., 2008; Angelis et al., 2014). In mammalian cell lines, FCF treatment causes highly intertwined and more densely packed, abnormally large septin structures, leading to reduced transmigration ability through small pores in transmigration assays (Hu et al., 2008). In another model with malignant mesothelioma cells from man and mice, treatment of respective cells with FCF resulted in accumulation of septin 7 structures along the plasma membrane, affecting the organization dynamics of septin filaments (Blum et al., 2019). Although we could not detect such decreased migration ability in FCF-treated cells (Figure 5), FCF treatment of primary cells used in our study similarly increased septin 7 density at the cell membrane and additionally triggered a shift of septin 7 structures to perinuclear regions (Figures 6I–L), which was even more prominent after cells had migrated (Figures 6M–P). Interestingly, this effect also occurred in untreated cells (Figures 6E–H). Surprisingly and in contrast to published data, FCF treatment did not only affect septin structures but had similar effects on localization of F-actin, which underlined the role of direct correlation and interaction of structures, and vice versa. These findings indicate septin 7 and F-actin redistribution away from the cell membrane in favor of enhanced cell plasticity in the course of migration, supporting respective proteins as key elements in migratory processes through remodeling of the cell cytoskeleton, as also shown in human T cells (Dupré et al., 2015).

Apart from changes in the cell membrane, these structural dislocations might also lead to enhanced adaption and reshaping of the cell's nucleus, since immune cells have to pass different confinements and mechanical barriers, sometimes even smaller than the cell itself (Renkawitz et al., 2019). While the cytoplasm, the plasma membrane, and small organelles can be easily adjusted, the cell's nucleus is the limiting factor due to its size and stiffness (Liu et al., 2016; Calero-Cuenca et al., 2018). This leads to the presumption that autoreactive PBLs from ERU cases have increased abilities to deform their cellular shape and nucleus to squeeze and fit through narrow pores, enabling invasion of the inner eye through the BRB. Relocation of septin 7 and F-actin to perinuclear regions, which we detected after cells had migrated (Figure 6), might have an impact on these processes. Therefore, the question of how cytoskeletal components interfere with and possibly regulate nuclear dynamics in order to deform and reduce the nuclear stiffness and rigidity still needs further clarification.

While interpreting our data, we have to keep in mind that *in vitro*-models have limitations to some extent, especially since cells are investigated outside of their natural surroundings, prohibiting interaction with other cell types which would usually occur. However, as we pursue to create more *in vivo*-like experimental conditions to increase reliable mechanistic insight into the pathogenesis of ERU, we first needed fundamental knowledge on migratory behavior and regulatory mechanisms, which we provide in the present study. The meaning of divergent migratory behavior of PBLs and pathogenesis-associated regulatory mechanisms in ERU and its translational value for autoimmune uveitis in man, however, needs to be assessed in further studies.

## Data Availability Statement

The raw data supporting the conclusions of this article will be made available by the authors, without undue reservation, to any qualified researcher.

## Ethics Statement

No experimental animals were used in this study. Collection of blood was permitted by the local authority, Regierung von Oberbayern (Permit number: ROB-55.2Vet-2532.Vet_03-17-88).

## Author Contributions

CD conceived, designed, analyzed the experiments, and supervised the project. CW and BA performed the experiments and analyzed the data. CD, CW, and RD analyzed the data. TW contributed samples and performed the experiments. CW and CD wrote the manuscript. All authors critically read the manuscript and approved the final version to be published.

### Conflict of Interest

The authors declare that the research was conducted in the absence of any commercial or financial relationships that could be construed as a potential conflict of interest.
