# Supplementary figures and images for: Aberrant Migratory Behavior of Immune Cells in Recurrent Autoimmune Uveitis in Horses
Source: Front Cell Dev Biol. 2020 Mar 10;8:101. doi: 10.3389/fcell.2020.00101 (PMC7076317; doi:10.3389/fcell.2020.00101)

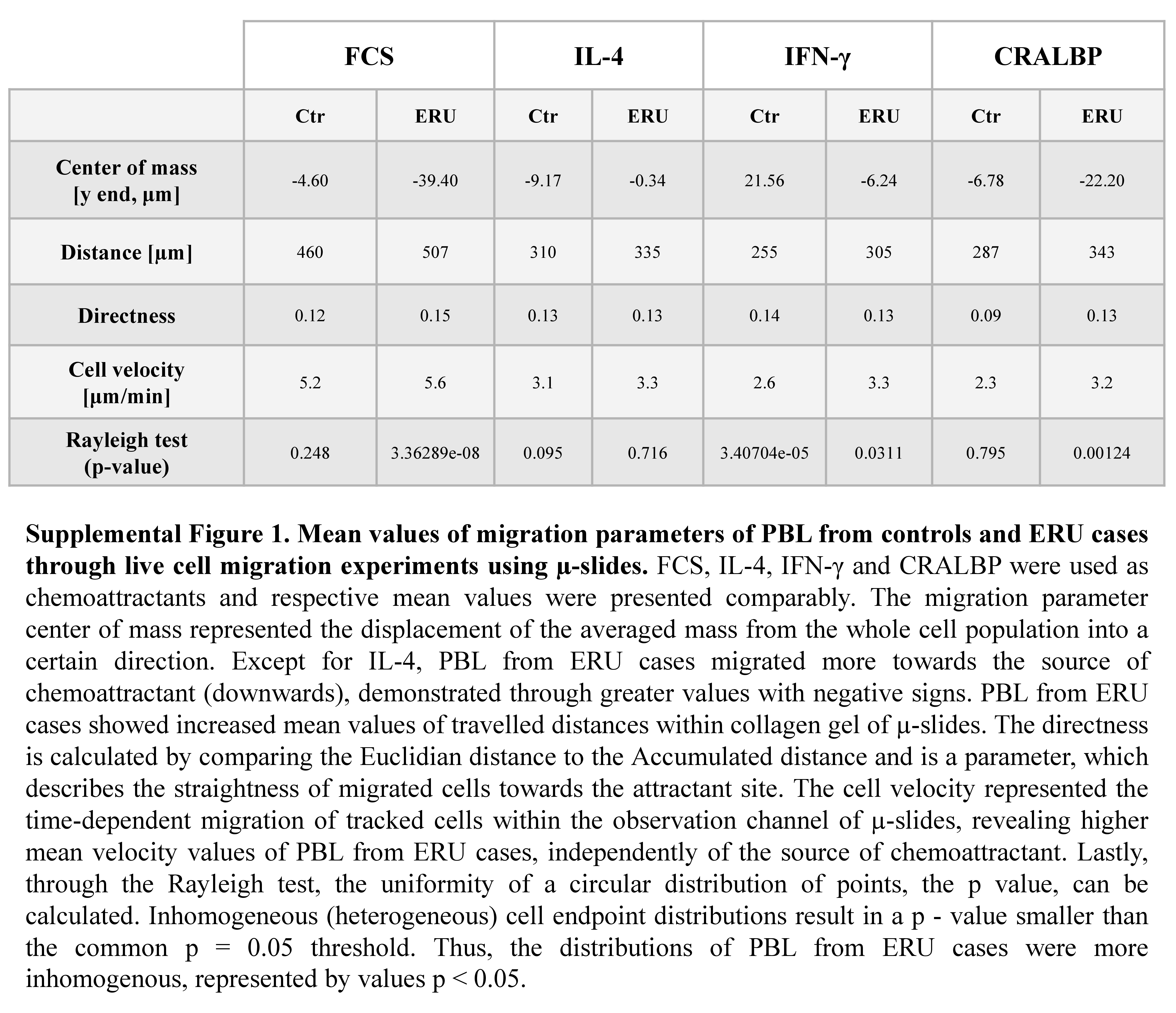

Supplement: Supplementary file 1 [file Image_1.TIF]

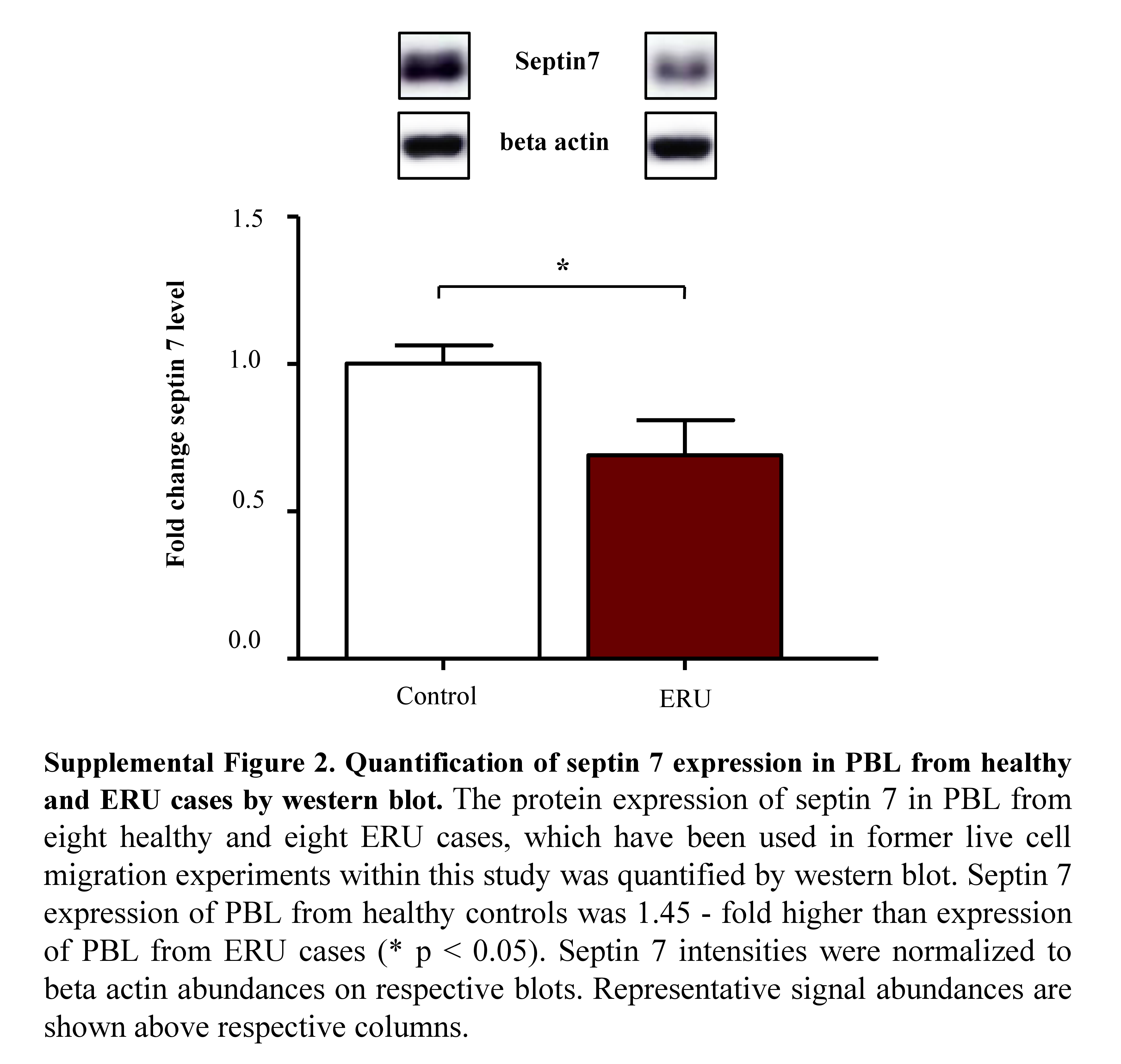

Supplement: Supplementary file 2 [file Image_2.TIF]
